# Patient-reported outcome (PRO) instruments used in patients undergoing adoptive cell therapy (ACT) for the treatment of cancer: a systematic review

**DOI:** 10.1186/s13643-023-02337-8

**Published:** 2023-09-30

**Authors:** Sally Taylor, Kate Law, Jake Coomber-Moore, Michelle Davies, Fiona Thistlethwaite, Mel Calvert, Olalekan Aiyegbusi, Janelle Yorke

**Affiliations:** 1 Christie Patient Centred Research, The Christie NHS Foundation Trust, Wilmslow Road, Manchester, M204BX UK; 2 School of Nursing and Midwifery, The University of Manchester, Oxford Road, Manchester, M13 9PL UK; 3 The Christie NHS Foundation Trust, Wilmslow Road, Manchester, M204BX UK; 4 The University of Manchester, Oxford Road, Manchester, M13 9PL UK; 5 Centre for Patient Reported Outcome Research (CPROR), Institute of Applied Health Research, University of Birmingham, Birmingham, UK; 6 National Institute for Health Research (NIHR) Applied Research Centre (ARC) West Midlands, Birmingham, UK; 7 Birmingham Health Partners Centre for Regulatory Science and Innovation, University of Birmingham, Birmingham, UK; 8 National Institute for Health Research (NIHR) Birmingham Biomedical Research Centre, Birmingham, UK; 9 Surgical Reconstruction and Microbiology Research Centre, National Institute for Health Research (NIHR), University of Birmingham, Birmingham, UK; 10 Midlands Health Data Research UK, Birmingham, UK; 11 DEMAND Hub, University of Birmingham, Birmingham, UK

**Keywords:** Adoptive cell therapy (ACT), Cancer, Patient-reported outcomes (PROs), Systematic, Review, Quality of life

## Abstract

**Introduction:**

Adoptive cell therapy (ACT) is a rapidly evolving field. Patient-reported outcomes (PROs) allow patients to report the impact of treatment on their quality of life during and after treatment. The systematic review aims to characterise the breadth of PROs utilised in ACT cancer care and provide guidance for the use of PROs in this patient population in the future.

**Methods:**

A systematic search was conducted (MEDLINE, PsycINFO, Embase and CINAHL) in August 2021 by two reviewers. Search terms covered the following: “adoptive cell therapy”, “patient-reported outcomes” and “cancer”. Studies were included if they used a PRO measure to report the impact of ACT. The methodological quality of PROs was assessed. Forward and backward reference searching was conducted of any relevant papers. A quality grading scale was applied based on Cochrane and Revenson criteria for classification of high-quality studies. Key data from the studies and the included PROs was extracted by two researchers and tabulated.

**Results:**

One-hundred nine papers were identified; 11 papers were included. The majority of studies were single-arm trials or observational studies. Twenty-two different PROs were identified; none was ACT specific. The PROMIS-29 and EQ-5D were most commonly used. Few studies collected PRO data in the first 1–2 weeks. Four studies followed patients up for over a year, and a further four studies followed patients for approximately 3 months.

**Discussion:**

None of the PROs identified have been designed specifically for ACT. Appropriateness of existing instruments should be considered. It should be considered whether it is appropriate to collect data more frequently in the acute stage and then less frequently during follow-up. It should be considered if one tool is suitable at all time points or if the tool should be adapted depending on time since treatment. More research is needed to identify the exact timings of PRO assessments, and qualitative work with patients is needed to determine the most important issues for them throughout the treatment and follow-up.

## Background

Surgery, chemotherapy and radiotherapy have been the main treatments for cancer for many years. More recently, however, new treatments such as targeted therapies and immunotherapies have started to be developed and have shown promising results in terms of survival [1]. Adoptive cell therapy or adoptive cell transfer (ACT) is a form of immunotherapy which harnesses the natural ability of immune cells to specifically recognise and eliminate target cells; thus, tumour-specific T cells can be infused to patients with the aim of the host immune system recognising and attacking cancer cells [2]. ACT has quickly become one of the fastest-growing areas of immune-oncology (IO) clinical research in the world [3]. ACT could be an additional treatment option for patients where other targeted and immunotherapy approaches have failed [4].

There are many types of ACT [5], but chimeric antigen receptors cell therapy (CAR T-cell) has made the most progress in terms of both clinical development and regulatory approval [6]. CAR T-cell treatments which target CD19 have demonstrated significant clinical benefit in clinical trials of patients with CD19 positive B-cell malignancies. A systematic review and meta-analysis of CAR-T trials found that CAR-T had better survival outcomes for large B-cell lymphoma patients compared to high-dose chemotherapy or autologous stem cell transplant [7]. It has been reported that children with aggressive lymphomas had very few treatment options available to them before CAR-T developments [1].

In 2017, the first two CD19-directed CAR-T therapies, tisagenlecleucel and axicabtagene ciloleucel, were approved by the Food and Drug Administration (FDA) and subsequently the European Medicines Agency (EMA) to treat patients with diffuse large B-cell lymphoma (DLBCL) and B-cell acute lymphoblastic leukaemia (B-ALL). Both of these licenced treatments are now being widely used in the commercial setting [8]. Since 2017, the haematological indications for approved CD19-directed CAR T-cell therapies have broadened to include conditions such as mantle cell lymphoma and certain types of follicular lymphoma, and in 2021, the first B-cell maturation antigen (BCMA)-directed CAR-T, idecabtagene vicleucel, was approved by the FDA for use in multiple myeloma. Tisagenlecleucel is another form of ACT which is also licensed for use. Since ACT is an emerging and expanding field, many treatments are still only available within the context of early phase clinical trials. Therefore, detailed knowledge of specific toxicities and in particular long-term effects may still be unknown. There is no consensus on what constitutes short- and long-term effects in this context; in a review of late effects, Chakraborty et al. [9] define toxicities that occur beyond 1–3 months post infusion as long-term effects, suggesting that short-term effects would occur in the first 3 months.

Due to the unique way ACT therapies work, many of the toxicities experienced by patients differ from those linked to traditional cytotoxic drugs and immunotherapies. CAR T-cell toxicities or the so-called on-target effects are variable, and the spectrum of toxicities depends on the specificity of the precise antibody target and T-cell activation [10]. Not all ACT treatments will have the same severity or range of toxicities. As safety data continues to emerge and mature, the toxicity profiles for these novel therapies will develop allowing more tailored patient management depending on the particular type of ACT therapy received. The toxicity profile for CD19 CAR T-cell is the most mature with over 1000 patients treated on CD19 CAR T-cell clinical trials in the USA alone, and increasing numbers of patients now treated both on and off trials globally [10]. Although even this group where toxicity data is more established, information on long-term events remains limited [11].

Acute toxicities are more well documented, and in fact, both cytokine release syndrome (CRS) and neurotoxicity, both of which have proved to have serious and/or life-threatening consequences, have received significant attention [12]. CRS is a spectrum of clinical and laboratory findings of fevers, hypotension, hypoxia and neurologic changes associated with substantial elevations of serum cytokine levels. The time of onset of CRS can be variable, ranging from a few hours to over a week after CAR T-cell infusion [13]. Neurologic toxicity is the second major side effect which has been seen in a substantial number of patients treated with CD19-targeted CAR T-cells. This toxicity is now widely referred to as immune effector cell-associated neurotoxicity syndrome (ICANS) [14] as it has also been observed following other types of cellular therapies. The clinical presentation of ICANS can be varied and includes tremor, headache, encephalopathy (confusion or delirium), expressive aphasia, motor weakness, seizures, depressed level of consciousness and, rarely, diffuse cerebral oedema. The onset of ICANS is also variable and can occur as early as 1 day post treatment, although presentation tends to be later than CRS and can occur up to the third or fourth week after infusion [13].

Patients can report toxicities they are experiencing using patient-reported outcomes (PROs). PROs are increasingly being used to ensure that patients have the opportunity to accurately report their own experience during and after treatment and provide a greater understanding of the impact of a disease and/or its treatments including any toxicities experienced [15, 16]. In clinical practice, PRO data can be used to inform and guide patient-centred care and clinical decision-making. In clinical trials, PROs can be used to assess the efficacy, safety and tolerability of treatments from the patient perspective, providing valuable insights into patients’ symptoms and health-related quality of life (HRQOL) [17, 18] and can be used to inform regulatory decision-making, clinical guidelines and health policy [19–22]. Evaluating PRO data alongside other trial outcomes can provide important information to aid in the understanding of the risks and benefits of treatment from the patients’ perspective. A Medicare Evidence Development and Coverage Advisory Committee (MEDCAC) meeting included a discussion with key stakeholders and experts in the field on the use of PROs in CAR-T trials. The panel emphasised the additional valuable data PROs provide but voiced concerns about their use due to lack of standardisation in scoring and patient and clinician understanding of the measures. These concerns need to be addressed to fully embrace the use of PROs in CAR T-cell trials.

It is important to consider the timing of PRO assessments within advanced cell therapy trials. Lasiter et al. (2020) recommend both short- and long-term follow-up. Regular PRO assessment should occur during the active treatment stage but given the curative expectations of these therapies, long-term follow-up (potentially up to 15 years for some trials) is recommended to identify any long-term side effects. Despite these treatments being in their infancy and the changing profile of toxicity overtime, it is necessary to further understand the short- and long-term effects of ACT. It is the aim of the potential lack of studies exploring long-term effects of ACT; it is still the aim within this review to include studies focusing on both short- and long-term effects.

Recent reviews have explored the collection of HRQOL data [23] or the use of PROs in CAR T-cell trials specifically [24]. Messina et al. [25] reported that the use of PROs in CAR T-cell trials is considerably less than the industry average (6.17% compared with 27%), despite the growing importance of HRQOL and its impact on value-based care [26–28]. The authors concluded that despite the increase in interest in ACT trials, there continues to be a deficiency of including and reporting of PROs in the trial design of this new therapeutic area. Similarly, Raymakers et al. [23] identified 424 CAR-T trials, and only 29 (6.8%) included HRQOL as a primary or secondary outcome.

Qualitative studies exploring the experience of patients receiving ACT treatment are limited. The three recent studies in this area have all focused on CAR-T rather than ACT more broadly [29–31]. Cheng et al. [29] reviewed a number of PROs that had been used in CAR-T trials and explored whether their study participants endorsed the items covered in these questionnaires. They reported that physical, emotional, social, role functioning and fatigue were all highly endorsed by patients, and cognitive function, pain, sleep, and general symptoms were moderately endorsed. They concluded that the EORTC-QLQ-C30 had the best coverage of items, but a CAR-T-specific questionnaire may be required to cover some of the key symptoms. Whisenant et al. [31] created a list of the commonly reported CAR-T treatment side effects, the most common being fatigue. Similar to Cheng et al. [29], Whisenet et al. [31] found that existing questionnaires did not cover all areas of importance of this patient group, and as a result, suggested adding additional items to the MDSAI to create the MDSAI-CT, a tool specific for patients receiving cellular therapy. In Jenei et al.’s qualitative study [30], they reviewed an online social media forum, and their findings focused on patient experienced more broadly such as accessing and navigating treatment and overcoming uncertainties.

Since ACT is a rapidly evolving field in cancer care with the potential to offer some patients durable response, it is important to understand what the short- and long-term effects of these novel treatments are for patients living with and beyond cancer. PRO data could inform the effective management of both short- and long-term physical and psychosocial morbidities.

The aim of this systematic review was to characterise the breadth of PROs utilised in ACT cancer care and provide guidance for the use of PROs in this patient population in the future. Specific objectives were as follows:

- Identify which PROs have been used in the ACT patient population in clinical trials or in clinical practice.
- Determine which instruments have been developed and validated in the ACT patient population.
- Summarise the available reliability and validity data and current use for PROs in the ACT patient population.
- Explore what issues are covered in PROs used in the ACT population.
- Identify any areas for future research in the use of PROs in ACT patients.

## Methods

### Search strategy

A systematic search was conducted on four databases (MEDLINE, PsycINFO, Embase and CINAHL) in August 2021. An information specialist helped to develop the search strategy. We used similar keywords across the databases, adapting the Boolean operators and MeSH vocabulary. The search terms used were related to the following: “adoptive cell therapy”, “patient-reported outcomes” and “cancer”. As the majority of ACT treatment is given in the context of clinical trials, it is anticipated that many of the studies identified will be clinical trials. We did however want to include as many relevant papers as possible; therefore, the search was not limited to clinical trials. Any studies including ACT patients in a clinical trial or clinical practice context were included. An example of the full research strategy can be found in Table 1. Results were not restricted by date or language. Articles identified were stored and managed in EndNote and duplicates removed.

**Table 1:** Example of full search terms used in MEDLINE

	**Ovid MEDLINE**	
1	exp Immunotherapy, Adoptive/	10,544
2	("Adoptive cell" adj2 (Therap* or Transfer*)).ab,kw,ti	1737
3	"Immune Effector Cell* ".ab,kw,ti	2095
4	"CAR-T Cell Therapy".ab,kw,ti	1822
5	1 or 2 or 3 or 4	14,529
6	Patient Reported Outcome Measures/	9141
7	(("Patient Reported" or "Patient-Reported") adj2 ("Outcome*" or "Measure*" or "Recovery" or "experience*")).ab,ti	24,352
8	(("patient reported treatment" or "patient-reported treatment") adj2 ("Outcome*" or "Measure*" or "Recovery" or "experience*")).ab,ti	24
9	(("self reported" or "self-reported") adj2 ("Outcome*" or "Measure*" or "Recovery" or "experience*")).ab,ti	11,101
10	(("self Reported Treatment" or "self-reported treatment") adj2 ("Outcome*" or "Measure*" or "Recovery" or "experience*")).ab,ti	27
11	(EPROs or EPRO or PROMs or PROM or EPROMS or EPROM).ab,ti	5305
12	("Patient*" and (PROs or PRO)).ab,ti	53,879
13	("Value based care" or "Value-based care").ab,ti	777
14	6 or 7 or 8 or 9 or 10 or 11 or 12 or 13	90,271
15	exp Neoplasms/	3,517,112
16	(tumour* or cancer* or neoplas* or oncolog* or malignan* or carcin* or metasta* or tumor* or mesotheliom* or sarcom* or lymphom* or leukaem* or leukem* or gliom*).ti,ab	3,947,418
17	("Acute b-cell lymphoblastic leuk?emia" or "B-ALL").ab,ti	5142
18	("Diffuse Large B-Cell Lymphoma" or "DLBCL").ab,ti	14,503
19	15 or 16 or 17 or 18	4,746,253
20	5 and 14 and 19	84

### Selection and screening

Identified articles were imported into the Rayyan systematic review reference system [32], and all were independently screened by two reviewers (S. T. and J. C. M.) against the inclusion and exclusion criteria. Studies were included if they used a PRO measure to report the impact of ACT treatment. There were no restrictions based on comparisons, outcomes assessed or study design. Initially, the titles and abstracts were screened. The full text was retrieved for any paper which potentially met the inclusion criteria. The two reviewers would discuss any discrepancies, and a third reviewer (M. D.) would be consulted if required. Forward and backward reference searching was used to identify any additional papers for inclusion.

### Data extraction

Two independent reviewers (S. T. and K. L.) used a structured Excel table to extract data from the selected full-text articles. Data collected included information regarding the publication (author(s), journal, year of publication), the study (type of study, setting), the intervention (ACT, treatment regimen), characteristics of the population (cancer type, stage, type of therapy) and outcomes. Data extraction also captured information related to the identified PRO measure(s) and aspects of clinical utility including name of the PRO, the concept it measured, mode and timing of administration, recruitment and completion rates and methodology used for the analysis (statistical or clinical significance, thresholds, type of measure and the method used for the analysis). Once the list of included papers had been generated, a list was created of the issues covered in the identified PROs these issues were cross-referenced against the symptoms and functional issues identified in available qualitative studies [29, 31] to explore if any of the PROs fully address the needs of this patient group.

### Methodological assessment of PRO use

The consensus-based standards for the selection of health status measurement instruments (COSMIN) checklist is a tool designed to assess key psychometric qualities (validity, reliability and responsiveness to change) of PROs [33]. Given the anticipated dearth of PROs developed specifically for the ACT population, it was decided that the modified version of the COSMIN checklist as recommended by the FDA [21] would be applied to any PROs not specific to ACT, and the full COSMIN tool would be applied if any ACT-specific tools were identified. The key psychometric qualities assessed in the modified version include conceptual framework, reliability and content validity, construct validity and clinical relevance of score changes (Table 2). It was felt that this would be sufficient for any non-ACT-specific PROs. Original validation papers for the included PROs were examined to collect relevant information of psychometric properties. An additional search was carried out for each questionnaire to identify any additional studies where the questionnaires had been used in the ACT population. Given that no RCTs were identified in the review, it was not felt appropriate to use the Cochrane risk-of-bias criteria. A description of the quality grading scale applied is presented in Table 3. This classification system was devised using Cochrane [34] and Revenson [35] criteria for classification of high-quality studies and has been used.

**Table 2:** Modified version of COSMIN checklist

Psychometric qualities	Description
Conceptual framework	All papers were examined to determine what details were presented about the constructs included within the measure
Reliability	Papers were explored to determine what reliability tests had been conducted including inter-item correlations, test–retest reliability and internal consistency
Content validity	Papers were explored to determine what level of construct validity in the form of interviews or focus groups had been conducted during the questionnaire development stages
Construct validity	Construct validity including whether the questionnaires could differentiate between particular clinical or demographic groups was explored
Clinical relevance of score changes	All data referring to the tool’s ability to detect changes over time were collected

**Table 3:** Study quality grading criteria (grade 1 indicates the highest quality study)

Quality grading criteria	
1	RCT with no methodological flaws
2	RCT with methodological flaws (validated questionnaires not used, insufficient sample size)
3	Randomised trial no control with no other methodological flaws
4	Randomised trial no control with methodological flaws (validated questionnaires not used, insufficient sample size)
5	Non-randomised controlled study with no methodological flaws
6	Non-randomised controlled study with methodological flaws (validated questionnaires not used, insufficient sample size)
7	One intervention group, no comparison

## Results

**Fig. 1:**
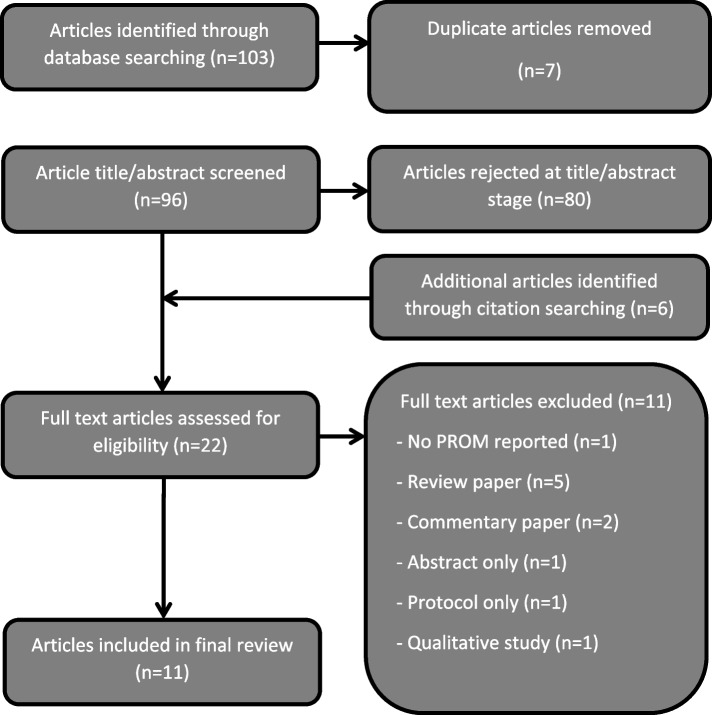
Flow diagram of articles identified for use in the review

The search retrieved 103 papers, seven duplicates were removed and the titles and abstracts of the remaining 96 were reviewed. A PRISMA flow diagram is presented in Fig. 1.

Sixteen papers were selected for full-text review, and a further six were identified following forward and backward reference searching. Of the 22 papers where full text was reviewed, 11 were excluded. Reasons for exclusion are as follows: no PRO reported (*n* = 1), review paper (*n* = 5), commentary paper (*n* = 2), abstract only (*n* = 1), protocol only (*n* = 1) and qualitative study (*n* = 1). References in the five review papers were searched to ensure all relevant papers had been included. Some of the included papers were published as abstracts only, but they included sufficient detail for data extraction. Searches were conducted to ensure full-text papers were not available. Given the limited number of eligible papers, it was decided that these papers would be included.

### Study characteristics

The majority of studies were conducted in the USA (*n* = 7) [36–42], three were international studies conducted in several countries [26, 43, 44] (see Table 4 for full list of countries included) and one was conducted in China [45] (Table 3). Six studies included participants with a single type of cancer: melanoma (*n* = 1) [45], myeloma (*n* = 2) [26, 38], leukaemia (*n* = 1) [43] and lymphoma (*n* = 2) [36, 44]. Two studies included participants with any haematological malignancy [39, 42], one study included lymphoma or leukaemia patients [37, 41] and one study included lymphoma, leukaemia or myeloma participants [40]. The number of participants included in the studies ranged from *n* = 16 to *n* = 128. The majority of studies were single-arm trials or observational studies and were graded as a 7 in terms of their methodological quality. Only two studies included a comparison arm, but these were not randomised controlled trials; one was an observational cohort study [39], and one was a retrospective study [45]. The majority of the trials used CAR-T treatment (*n* = 8) [36–42, 46], two used Tisagenlecleucel [43, 44] and one used adoptive immunotherapy using cytokine-induced killer (CIK) cells [45].

**Table 4:** Study characteristics

Author and year	Country	Cancer type	Sample size (consented)	Type of therapy	Trial design	PROs used	Timing of PRO assessments	Was at least one PRO validated?	Intervention effect	Quality grading
Hoogland et al. (2021) [36]	USA	Relapsed/refractory large B-cell lymphoma	103	CAR-T (axi-cel)	Observational	SF-36 **OR** PROMIS-29* and PRO-CTCAE	Toxicities baseline and 14, 30, 60 and 90 days after treatment QoL baseline and 90 days	Yes	Depression improved overtime but not significant	7
Knight et al. (2020) [37]	USA	Relapsed refractory B-cell non-Hodgkin lymphoma and chronic lymphocytic leukaemia	16	CAR-20/19 T cells	Single-arm intervention study	Inventory of depression and anxiety symptoms (IDAS), Brief Pain Inventory (BPI), Fatigue Severity Index (FSI), Pittsburgh Sleep Quality Index (PSQI)	Baseline, day 14/28/90 post-intervention	Yes	Depression improved overtime but not significant	7
Li et al. (2017) [45]	China	Melanoma	104	Adoptive immunotherapy using cytokine-induced killer (CIK) cells	Retrospective-two-arm CIK and conventional or conventional treatment only	Simplified questionnaire about QoL	Every 3 months for the first year and every 6 months onwards	No	Some reported improvements (appetite, sleep, weight gain, pain) but not statistically significant	6
Martin et al. (2020) [38]	USA	Multiple myeloma	68	CAR-T (ciltacabtagene autoleucel)	Single-arm intervention study	EORTC QLQ-C30 and multiple myeloma (MY20), EQ-5D-5L)	Baseline, 7, 28, 56, 78, & 100	Yes	Clinically meaningful improvements at day 100 (pain, fatigue, physical function, global health, thinking about illness, worries about future)	7
Mullane et al. (2020) [40]	USA	Relapse/refractory non-Hodgkin lymphoma (NHL), acute lymphoblastic leukaemia (ALL), chronic lymphocytic leukaemia (CLL) and multiple myeloma (MM)	58	CAR-T	Observational	PROMIS Scale v1.2 Global Health, PROMIS-29 Profile v2.1, 30 additional questions, including questions recognitive function	Single questionnaire	Yes	Clinically meaningful differences (global physical health, anxiety/depression)	7
Ruark et al. (2021) [41]	USA	Relapsed/refractory ALL, NHL, or CLL	40	CAR-T (CD-19)	Observational	PROMIS Scale v1.2 Global Health, PROMIS-29 Profile v2.1, 30 additional questions, including four questions recognitive function	Single questionnaire	Yes	No clinical meaningful differences in scores. Some scores for anxiety and/or depression and Global Mental Health indicated worse health than general population	7
Wang et al. (2021) [42]	USA	Haematological	60	CAR-T	Cross-sectional observational study	MD Anderson Symptom Inventory; PROMIS-29; EQ-5D-5L; single-item HRQOL; CAR T-cell therapy-specific symptoms	Single questionnaire	Yes	Observational only	7
Laetsch et al. (2019) [43]	International (Australia, Austria, Belgium, Canada, France, Germany, Italy, Japan, Norway, Spain, USA)	Relapsed or refractory B-cell acute lymphoblastic leukaemia	75	Tisagenlecleucel	Single-arm open-label phase 2	The Pediatric Quality-of-Life Inventory (PedsQL) and the European Quality-of-Life-5 Dimensions (EQ-5D) questionnaire	Day 28, then once a month for 6 months, then every 3 months until month 12, and then will be followed up for 5 years	Yes	Minimal clinically important differences observed from month three onwards	7
Shah et al. (2020) [47]	International (USA, Canada, Belgium, France, Japan, Germany, Italy, Spain)	Relapsed and refractory multiple myeloma	128	CAR-T (Ide-cel)	Single-arm interventional study	EORTC Quality of Life C30 (QLQ-C30) and Myeloma Module (MY20) questionnaires and EQ-5D-5L	Baseline, months 1–6, 9, 12 and 15 post-infusion	Yes	Clinically meaningful differences seen on specific subscales of each PRO	7
Sidana et al. (2019) [39]	USA	Haematological	93	CAR-T	Longitudinal observational cohort study	FACT-G, PROCTCAE, Neuro-QoL v2	Baseline, week 2, months 1,2, 3	Yes	Statistically significant difference in QoL at each time point	5
Maziarz et al. (2020) [44]	USA, Europe, Japan, Canada and Australia	Relapsed/refractory diffuse large B-cell lymphoma	115	Tisagenlecleucel	Single, open-label, phase 2 study	Function Assessment of Cancer Therapy-Lymphoma (FACT-Lym), SF-36	Baseline, months 3, 6, 12 and 18	Yes	SF-36 instruments showed improvement above the minimal clinically important differences on 5 of 8 subscales	7

^*^The questionnaire was changed during the study so earlier participants completed SF-36 and later participants completed PROMIS-29

### Patient-reported outcomes

A total of 23 different PROs were used across the studies (Table 5). The most commonly used PRO were the PROMIS-29 [48] which was used in four of the identified studies [36, 40–42] and the EQ5D [49] which was used in three studies [38, 42, 43]. Ten of the PROs were generic measures not focusing on any particular disease group. Seven of the generic PROs were measures assessing a broad range of different functional aspects such as mobility, activities of daily living and impact on social and emotional function. One of these generic tools was developed specifically for paediatric patients. Four of the generic questionnaires focused on a specific symptom (pain, anxiety and depression, sleep, cognition). A further one that could not be located appeared to focus on fatigue. Four PROs were cancer specific and measured a wide range of symptoms or functional issues. Three PROs were disease specific and focused on neurological diseases, myeloma and lymphoma. Five studies either use PROs specific to the included patient population or state that the questionnaires had been validated in the stated patient population. None of the questionnaires used had been developed specifically to collect PRO data for patients receiving ACT.

**Table 5:** Review of psychometric properties of identified questionnaires using modified version of COSMIN checklist

PRO	Study used in	Conceptual model	Reliability	Content validity	Construct validity	Responsiveness to change	Target population of PRO: generic, cancer, disease, or ACT specific
SF-36 Health Survey (SF-36) [50]	Hoogland, Maziarz	Eight multi-item dimensions covering functional status, wellbeing and overall evaluation of health	Internal consistency and test–retest demonstrated	Patient interviews conducted	Yes—scores distributed as expected for sex, age, social class, use of health services and for patients with chronic disease	Not mentioned	Generic
Patient-Reported Outcome Measurement Information System-29 (PROMIS-29) [48]	Hoogland, Mullane, Ruark, Wang	Assesses pain intensity using a single 0–10 numeric rating item and seven health domains (physical function, fatigue, pain interference, depressive symptoms, anxiety, ability to participate in social roles and activities, and sleep disturbance) using four items for each domain	Internal consistency. More reliable than existing summary scores	No details provided	No details provided	No details provided	Generic
Patient-Reported Outcomes-Common Terminology Criteria for Adverse Events (PRO-CTCAE) [51]	Hoogland, Sidana	PRO-based measurement system to capture symptomatic adverse events by self-report in cancer clinical trials	Test–retest reliability was acceptable for 36/49 pre-specified items	Patient interviews conducted	Overall, 119/124 items met at least one construct validity criterion	Statistically significant correlations were observed between PRO-CTCAE item changes and corresponding QLQ-C30 scale changes for all 27 pre-specified items (median *r* = 0.43, range 0.10–0.56; all *P* ≤ .006)	Cancer specific
Inventory of Depression and Anxiety Symptoms (IDAS) [52]	Knight	To create specific symptom scales reflecting distinctive aspects of depression and anxiety	Test–retest reliability figures ranged from 0.72 to 0.83. Good internal consistency	No details provided	Presented data correlating the IDAS with both the HRSD and the IMAS, we have not yet examined it in relation to formal DSM-IV diagnoses of major depression and the anxiety disorders	No details provided	Generic
Brief Pain Inventory (BPI) [53]	Knight	Measures sensory and reactive pain. Rate intensity and how much pain interferes with activities	Good internal consistency (*CA* 0.78–0.95 across the two scales). Test–retest reliability is mixed	Patient interviews conducted	Factor analysis was consistent across different clinical groups	Ability to detect clinically meaningful change	Generic
Fatigue Severity Index (FSI)	Knight	Questionnaire could not be located					
Pittsburgh Sleep Quality Index (PSQI) [54]	Knight	Aims to discriminate between good and poor sleepers and be a useful tool for researchers and clinicians. Assesses sleep duration and latency and frequency and severity of sleep problems	Good test–retest reliability	Developed using experience with patients but interviews not mentioned	Significant differences across groups	Not mentioned	Generic
Simplified QoL questionnaire	Li	None	None	None	None	None	Study specific
European Organisation for Research and Treatment of Cancer Quality of Life Core 30 (EORTC QLQ-C30) [55]	Martin, Shah	Generate a core questionnaire incorporating a range of physical, emotional and social health issues relevant to a broad spectrum of cancer patients, irrespective of specific diagnosis. This core instrument could then be supplemented by diagnosis-specific (e.g. lung cancer or breast cancer) and/or treatment-specific questionnaire modules	The recommended 0.7 for good internal consistency between groups was met for 8 of the 9 subscales	Patient interviews	Could discriminate across clinical criteria	Significant changes in the right direction were reported for functional scales	Cancer specific
European Organisation for Research and Treatment of Cancer Multiple Myeloma (EORTC MY20)	Martin, Shah	To assess the disease-specific symptoms of myeloma and their impact on everyday life and treatment-related issues, mainly side effects of chemotherapy. To be used in conjunction with QLQ-C30	Internal consistency was greater than 0.7 CA for all scales	Interviews with patients, oncologists and haematologists	Correlations with QLQ-C30 items. Two subscales (disease symptoms and side effects) and the body image item could discriminate by PS and patients with/without fractures	Pain was only scale to show significant change over time	Disease specific (myeloma)
EuroQoL 5D (EQ-5D-5L) [56]	Martin, Wang, Shah	Generic instrument for describing and valuing health	Korean version reliable in cancer patients	Patient interviews	Scores were reported in expected direction for key characteristics, e.g. age, education, smoking, status	Could detect improvements and deterioration in health (breast cancer)	Generic
EuroQoL 5D EQ-5D-3L [57]	Laetsch	A standardised non-disease-specific instrument for describing and valuing HRQOL			Responses conform to what would be expected for key characteristics		Generic
EuroQoL 5D Youth (EQ-5D-Y) [58]	Laetsch	A standardised non-disease-specific instrument suitable for children and adolescents	Test–retest results were good for most domains. Ceiling effects for mobility and self-care	Interviews with healthy and chronically ill young people	High correlations with existing questionnaires. Able to distinguish between those with chronic pain and those without	Largest treatment effect observed in chronically ill children. Poorer responses in children with minimal pr no health concerns	Generic
Patient-Reported Outcome Measurement Information System Global Health (PROMIS Global Health) [59]	Mullane, Ruark	Global health refers to a person’s general evaluations of health rather than any of its specific components. The global health items include global ratings of the five primary PROMIS domains (physical function, fatigue, pain, emotional distress and social health) and general health perceptions that cut across domains			Correlations with comparable items from PROMIS		Generic
Additional questions	Mullane, Ruark	None	None	None	None	None	Study specific
MD Anderson Symptom Inventory (MDASI) [60]	Wang	Brief measure of the impact and severity of symptom items	The values of a for the two sets of symptom items and the interference scales, respectively, were 0.85, 0.82 and 0.91 for the validation sample and 0.87, 0.87 and 0.94 for the cross-validation sample, which shows a high level of reliability for these sets of items	Clinician assessment but patients not mentioned	Able to differentiate between PS	Not mentioned	Cancer specific
Single-item HRQOL	Wang	None	None	None	None	None	Study specific
CAR T-cell therapy-specific symptoms	Wang	None	None	None	None	None	Study specific
The Pediatric Quality-of-Life Inventory (PedsQL) [61]	Laetsch	Integrates generic core scales and disease-specific modules into one measurement system. Designed to measure core health domains covered in WHO	Most self-report scales and proxy-report scales approached or exceeded the minimum reliability standard of 0.70	No details provided	The PedsQL performed as hypothesized utilising the known-groups method. The PedsQL differentiated HRQOL between healthy children and those with acute or chronic health conditions and was correlated with measures of morbidity and illness burden. The MTMM analyses tested convergent and discriminant validity across methods. The heterotrait-monomethod analyses are consistent with the conceptualization of the PedsQL as measuring an integrated multidimensional construct	No details provided	Generic (paediatric)
Functional Assessment Cancer Therapy-General (FACT-G) [62]	Sidana	Generic scale which can be combined with disease-specific modules. Quality of life treated as a subjective multidimensional concept	Good internal consistency demonstrated for the subscales	Patient interviews used to generate items	Convergent and divergent validity were demonstrated when compared with other measures. Able to differentiate between stage of disease	Could detect change over time in performance status	Cancer specific
Quality of Life in Neurological Disorders (Neuro-QoL v2) [63]	Sidana	Neuro-QoL is a new, standardized approach to measuring HRQL across common neurologic conditions		Patient-focus groups		Conditional minimal detectable change scores have been estimated for Neuro-QoL short forms. Thresholds for severity of four Neuro-QoL measures (fatigue, upper extremity function, lower extremity function-mobility, sleep disturbance) have been estimated using a modified bookmarking methodology based on the perspective of individuals with multiple sclerosis and clinicians	Disease specific (neurological)
Functional Assessment of Cancer Therapy (FACT-Lym) [64]	Maziarz	Lymphoma-specific questionnaire designed to compliment FACT-G	Internal consistency coefficients for the 15-item LymS (0.79, 0.85 and 0.84 T1–T3) and test–retest stability (0.84) indicated good reliability	Interviews with clinicians and patients	Did not differentiate between patient groups defined by NHL grade. Patients currently on treatment had lower FACT-Lym scores. Moderate correlations with POMS, SF-36 and PCS	Able to differentiate between three patient groups overtime (worse unchanged better)	Disease specific (lymphoma)

Given that none of the identified PROs had been developed specifically for ACT patients, we felt it was important to examine whether the PROs covered the experiences of this patient group. Two qualitative studies [29, 65] were identified which explored the QOL experiences of a total of 39 ACT patients. The issues identified in these studies were tabulated and then cross-referenced with the items covered in all the PROs included in this review. None of the included PROs covered all items identified in the two qualitative research studies (Table 6). The PRO-CTCAE had the best coverage of items, but there were still some areas that were not addressed. The majority of studies used multiple PROs in a single study. The quality of the included PROs was mixed, some were well validated whereas others had been developed specifically for the study and had not been subjected to any psychometric testing. All but one study [45] used at least one validated PRO. The timing of PRO administration varied across the studies, some were completed at a single time point only (*n* = 3) [40–42] whereas other studies included multiple time points. There was no consistency in the time points used, only one study collected PRO data at 7 days [38] and three collected it at 14 days [36, 37, 39].

**Table 6:** Item coverage in identified PROs cross-referenced with issues identified by at least 2 participants in a qualitative study exploring experiences of CAR-T [31]

PRO	Physical function	Emotional function	Role function	Social function	Fatigue	Pain	Cognitive	Sleep	Appetite	Headache	Chills/cold	Light headedness	Swelling	Bowel issues	Nausea/vomiting	Shaking hands	Coughing
SF-36 Health Survey (SF-36) (Brazier et al., 1992) [50]	X	X	X	X	X	X											
Patient-Reported Outcome Measurement Information System-29 (PROMIS-29) (Hays et al., 2018) [48]	X	X	X	X	X	X		X									
Patient-Reported Outcomes-Common Terminology Criteria for Adverse Events (PRO-CTCAE) (Dueck et al., 2015) [51]		X			X	X	X	X	X	X	X	X	X	X	X		X
Inventory of Depression and Anxiety Symptoms (IDAS) (Watson et al., 2007) [52]		X															
Brief Pain Inventory (BPI) (C. Cleeland & Ryan, 1994) [53]	X	X	X	X		X		X									
Fatigue Severity Index (FSI)					X												
Pittsburgh Sleep Quality Index (PSQI) (Buysse et al., 1989) [54]		X		X		X		X			X						X
Simplified QoL questionnaire						X		X	X								
European Organisation for Research and Treatment of Cancer Quality of Life Core 30 (EORTC QLQ-C30) (Aaronson et al., 1993) [55]	X	X	X	X	X	X	X	X	X					X	X		
European Organisation for Research and Treatment of Cancer Multiple Myeloma (EORTC MY20)	X	X	X	X	X	X	X	X	X					X	X		
EuroQoL 5D (EQ-5D-5L) (Herdman et al., 2011) [56]	X	X	X	X		X											
EuroQoL 5D EQ-5D-3L (Rabin & De Charro, 2001) [57]	X	X	X	X		X											
EuroQoL 5D Youth (EQ-5D-Y) (Wille et al., 2010) [58]	X	X	X	X		X											
Patient-Reported Outcome Measurement Information System Global Health (PROMIS Global Health) (Hays et al., 2009) [59]	X	X	X	X	X	X											
PROMIS Cognitive Function (Mullane)							x										
Additional questions																	
MD Anderson Symptom Inventory (MDASI) (C. S. Cleeland et al., 2000) [60]		x			x	x	x	x	x						x		
Single-item HRQOL																	
CAR T-cell therapy-specific symptoms		x			x	x	x				x	x	x			x	x
The Pediatric Quality-of-Life Inventory (PedsQL) (Alderfer & Marsac, 2013) [61]	x	x	x	x													
Functional Assessment Cancer Therapy-General (FACT-G) (Cella et al., 1993) [62]	x	x	x	x	x	x		x							x		
Quality-of-Life in Neurological Disorders (Neuro-QoL v2) (Gershon et al., 2012) [63]							x										
Functional Assessment of Cancer Therapy (FACT-Lym) (Hlubocky et al., 2013) [64]	x	x	x	x	x	x	x	x	x						x		

Four studies followed patients up for over a year [43–45, 47], with the longest follow-up period being 5 years [43]; a further four studies followed patients for approximately 3 months [36–39]. The completion rates for most studies were above 70%; three studies however had very low response rates (44%; 38%; 18%). The majority of studies do not provide details as to how the PRO was administered. The three studies where details were provided were administered online; one of these studies also had a paper option if required.

Although only two studies included a comparison arm [45], the majority of studies reported on whether there was a significant difference in their outcome measures. Only one study [39] reported a statistically significant difference in PRO responses. Sidana et al. reported that the CAR T-cell group experienced significantly less worsening in quality of life as measured by FACT-G than both autoSCT and alloSCT groups from baseline to each time point (week 2, months 1, 2 and 3) [39]. Five studies reported minimal clinically important differences in some elements of the PRO data [38, 40, 43, 44, 47].

## Discussion

Eleven studies were identified in this review, the majority of which were conducted in the USA. A relatively low number of ACT studies were identified that collected PRO data. In comparison to the number of studies, a large number of PRO instruments were identified which highlights the need for further research to standardise assessment in this area. Measuring patient experience and patient-reported outcomes is crucial in the development of new treatments. PRO toxicity data has been reported to be more accurate than clinician-reported data [66, 67]. As expected given the focus of ACT clinical trials [68], the majority of studies only included patients with haematological cancer. Only one study [39] reported a statistically significant difference in PRO responses; the CAR T-cell group experienced significantly less worsening in QoL as measured by FACT-G than both stem cell transplant groups.

The studies included in this review have highlighted many methodological challenges to the use of PRO data in the ACT population. The wide range of PROs used make it difficult for any comparisons to be made across the data collected. As highlighted in other research, the majority of studies do not use the PRO as their primary outcome [69]. The majority of ACT trials are early phase with safety as a priority; therefore, investigators may be reluctant to include QoL as an outcome [23]. The study designs were also not methodologically robust, no RCTs were included and only two included a comparison arm. This is a problem unique to ACT as traditionally ACT studies would generally be tested in early phase trials only. RCTs are the gold standard in clinical trials, and RCT data would be needed to fully explore the impact of ACT on toxicities and quality of life. The type of patients included is also a concern as some of the studies only report results on the more well patients or have a significant dropout rate; therefore, the results may not be generalisable. Furthermore, the needs of underserved populations may not have been considered [70].

Choosing an appropriate PRO instrument is a crucial step when developing a trial. Ideally, a PRO should be valid, reliable, sensitive to change and be generalisable to the target patient population [71]. PROs can be generic or disease specific. Many of the cancer PROs aim to assess quality of life and key symptoms or toxicities. None of the studies identified in this review uses PROs designed specifically for the ACT population. Further research is required to understand the concepts that matter to patients undergoing ACT. In their qualitative study, Whisenant et al. [65] explored the symptom burden of CAR T-cell therapy. Participants reported experiencing 34 symptoms related to the disease and treatment; they also described how their symptoms interfered with their functioning in many different areas. In this review, we cross-referenced qualitative findings with the issues covered in the identified PROs and found that none of the PROs covered all the issues pertinent to this patient group. This is perhaps why many of the included studies used multiple questionnaires. There is a danger however of overburdening patients if they are expected to complete large numbers of questionnaires. More qualitative research is needed to further explore experience in a wider population of ACT patients. Qualitative findings can be used to inform the creation of new PROs where required.

The timing of PRO assessments is an important issue that needs to be considered [69]. Lasiter et al. suggest there are three distinct phases in PRO data collection: acute, sub-acute and long term; they suggest that timing of PRO assessments should vary within these phases, and different PROs may also be used in order to address the most pertinent issues at each time point [72]. The acute phase when patients are likely to experience toxicities is considered to be weeks 1–4, and it is recommended that PRO data should be collected at least once a week during this phase [73]. Some of the key toxicities such as CRS and ICANS are likely to occur 1–2 weeks after treatment. Only one of the included studies collected PRO data at 7 days, and three collected it at 14 days. The majority of studies therefore may not have collected data on the key toxicities patients may experience. It is important to monitor toxicities in new therapeutic areas as these toxicities could be predictors of worse complications in the future. One qualitative study [29] suggested that the majority of patient side effects had improved after 6 months. After this time point, the emphasis of collecting data may shift to more general QoL rather than PROs that collect information on symptoms.

The follow-up period varied across the included studies. Four studies followed patients up for over a year, with the longest follow-up period being 5 years; a further four studies followed patients for approximately 3 months. Long-term follow-up is important with a relatively new drug as data needs to be collected on long-term toxicities. Existing research has not explored the role of collecting of PRO data overtime [69]. There are practicality issues with following patients up for a long time however as their care may be transferred to other centres, and therefore, keeping track of patients and getting clinical teams to manage and take ownership of follow-up may be difficult [73]. Electronic patient-reported outcomes (ePROs) are becoming more common place and may make longer-term follow-up more feasible [46]; however, issues of digital inclusion must be considered [70]. There is also the issue of patient compliance. In some studies included in this review, compliance rates were very low. Whatever time points are selected, it is important to ensure that baseline data is collected as the patients in this population may have received several lines of treatment before their ACT, and as a result, their baseline data may be significantly lower than some patient populations.

Attempts have been made to make recommendations for the most suitable questionnaires to be used in the ACT population. In this review, the most commonly used PROs were the PROMIS-29 which was used in four of the identified studies and the EQ5D which was used in three studies; however, frequency of use should not be the main driver for future practice. Rather this should be informed by addressing concepts that matter to patients and other key stakeholders. PROMIS, FACT-G and PRO-CTCAE were recommended in one review as possible PROs to use in this patient population [73] PROMIS was recommended due to its high correlation with SF36, ease of comparison with general population and free availability. FACT-G was recommended due to the availability of historical data in different cancer subtypes and PROCTCAE was recommended as a suitable measure of toxicity [73]. The Centers for Medicare & Medicaid Services (CMS) panel have also made their own recommendations about the most appropriate tools to use (PROCTCAE [51], MD Anderson Symptom Inventory [60], EORTC-QLQ-C30 [62] and PROMIS [48]), all of which are flexible questionnaire systems that use item banks or a modular approach. It is important to consider the purpose of the PRO tool as PROs are designed to measure different things. Some PROs focus on HRQOL, whereas others focus on toxicities; therefore, different PROs may be required at different points of the disease trajectory.

### Limitations

There are many methodological concerns with the studies identified in this review such as a lack of robust study designs and concerns about the patient population included; additionally, a wide range of PROs have been used across the studies. These issues make it difficult for any recommendations to be made about the appropriate PRO to be used in the ACT patient population. It was a concern that such a small number of papers were identified in the search. We did however work with information specialists to generate the search terms and also searched key authors and reference lists; therefore, we are confident that all relevant papers have been identified.

## Conclusion

None of the PROs identified has been designed specifically for the ACT population, and none of them covered all the needs of this patient population. Appropriateness of existing instruments should be considered and if necessary a new tool developed. It should be considered whether it is appropriate to collect data more frequently in the acute stage and then less frequently for a considerable time during the follow-up in order to identify late effects and whether one tool is suitable at all time points or if the tool should be adapted depending on time since treatment. More research is needed to identify the exact timings of PRO assessments, and qualitative work with patients is needed to determine what are the most important issues for them throughout the treatment and follow-up trajectory.

## Data Availability

All data generated or analysed during this study are included in this published article. The protocol was not published but can be obtained from the authors if required.
