# Correction: Patient‑reported outcome (PRO) instruments used in patients undergoing adoptive cell therapy (ACT) for the treatment of cancer: a systematic review

**DOI:** 10.1186/s13643-024-02540-1

**Published:** 2024-04-30

**Authors:** Sally Taylor, Kate Law, Jake Coomber‑Moore, Michelle Davies, Fiona Thistlethwaite, Mel Calvert, Olalekan Aiyegbusi, Janelle Yorke

**Affiliations:** 1 Christie Patient Centred Research, The Christie NHS Foundation Trust, Wilmslow Road, Manchester, M204BX UK; 2 School of Nursing and Midwifery, The University of Manchester, Oxford Road, Manchester, M13 9PL UK; 3 The Christie NHS Foundation Trust, Wilmslow Road, Manchester, M204BX UK; 4 The University of Manchester, Oxford Road, Manchester, M13 9PL UK; 5 Centre for Patient Reported Outcome Research (CPROR), Institute of Applied Health Research, University of Birmingham, Birmingham, UK; 6 National Institute for Health Research (NIHR) Applied Research Centre (ARC) West Midlands, Birmingham, UK; 7 Birmingham Health Partners Centre for Regulatory Science and Innovation, University of Birmingham, Birmingham, UK; 8 National Institute for Health Research (NIHR) Birmingham Biomedical Research Centre, Birmingham, UK; 9 Surgical Reconstruction and Microbiology Research Centre, National Institute for Health Research (NIHR), University of Birmingham, Birmingham, UK; 10 Midlands Health Data Research UK, Birmingham, UK; 11 DEMAND Hub, University of Birmingham, Birmingham, UK

**Correction:** **Syst Rev 12, 183 (2023)**

10.1186/s13643-023-02337-8

Following publication of the original article [1], there was an error in the author name ‘Fiona Thistlewaite’ which should have been ‘Fiona Thistlethwaite’. The original article has been corrected.
